# Validation of the new COPD assessment test translated into Thai in patients with chronic obstructive pulmonary disease

**DOI:** 10.1186/1471-2466-14-193

**Published:** 2014-12-04

**Authors:** Chaicharn Pothirat, Sumalee Kiatboonsri, Charoen Chuchottaworn

**Affiliations:** Division of Pulmonary, Critical Care, and Allergy, Department of Internal Medicine, Faculty of Medicine, Chiang Mai University, Chiang Mai, 50200 Thailand; Department of Medicine, Faculty of Medicine, Ramathibodi Hospital, Mahidol University, Bangkok, 10400 Thailand; Department of Medical Services, Central Chest Institute of Thailand, Ministry of Public Health, Nonthaburi, 11000 Thailand

**Keywords:** Chronic obstructive pulmonary disease, Health status assessment, Quality of life, Questionnaire, Validity

## Abstract

**Background:**

The COPD Assessment Test (CAT™) is a new questionnaire that has been developed recently for measuring the COPD patient’s health status. It is known to have a good correlation with disease specific health status measured by St. George’s Respiratory Questionnaire (SGRQ). For the wider application in clinical practice, it has been validated in many countries. We evaluated the reliability and validity of the translated CAT questionnaire for Thai COPD patients.

**Methods:**

The study was designed as a cross-sectional validation study enrolling stable Thai COPD patients from three academic centers in Thailand at a single visit. The original CAT questionnaire was translated to Thai through linguistic validation process. The official Thai CAT and SGRQ questionnaires were filled by Thai patients after orientation by the out-patient nurse. The reliability of all items was assessed by Cronbach’s formula for coefficient using pooled data from all patients. The validity of the questionnaire was tested using Pearson’s correlation with SGRQ.

**Results:**

A total of 98 Thai COPD patients completed the official Thai CAT questionnaire; 83% were male, mean age 71 years (SD 8.2), and % predicted of FEV_1_ 56.6% (SD 20.9). The official Thai CAT questionnaire was shown to have a high internal consistency (Cronbach's α coefficient = 0.853). The assessment of validity of official Thai CAT questionnaire was moderately correlated with that of SGRQ (r = 0.652).

**Conclusions:**

The official Thai CAT questionnaire has an acceptable reliability and validity. It can be expected to serve as a short and simple tool for assessment of the health status of Thai COPD patients.

## Background

Chronic obstructive pulmonary disease (COPD) is defined as a preventable and treatable disease with significant extra pulmonary effects that may contribute to the severity of the condition in many patients. COPD is a major health concern worldwide, and it has a substantial impact on a patient’s life. According to the treatment guidelines, the general approach to managing COPD should address improvement in quality of life (QoL) [1] which is defined as an individual’s perception of his life in the context of culture and values [2]. Many patients with COPD have impaired health related QoL [3] and low QoL is strongly associated with poor prognosis of COPD [4]. Lung function tests alone does not provide a measurement of the overall impact of COPD health status, even though they are used for classification of disease severity [5]. Effective dialogue between physicians and patients during consultation sessions can address the impact of COPD on a patient’s QoL. St. George’s respiratory questionnaire (SGRQ), the standard tool for assessment of QoL in COPD, is lengthy and time consuming [6]. The COPD Assessment Test (CAT) questionnaire developed recently in the English language, is a short and simple, self-administered questionnaire to assess the overall impact of COPD on patients, as well as to improve patient–physician communication [6]. It is known that the validation of the questionnaire might be influenced by languages, cultures, and ethnicities [7, 8]. Most of the validation findings so far have been based on data from English speaking countries, mainly in the United States and European countries. Our study may provide a better understanding of the CAT in the Thai language, ethnicity, and cultural group. The primary objective is to evaluate the reliability and validity of the official Thai CAT questionnaire. The secondary objectives are to examine relationships of CAT with forced expiratory volume in first second (FEV_1_) and Medical Research Council (MRC) dyspnea score.

## Methods

The study was conducted as a cross-sectional validation design with a single visit. All subjects were recruited from chest clinics of three academic hospitals in Thailand between April-August 2012. This study was approved by the Research Ethics Committee, Faculty of Medicine, Chiang Mai University; the Committee of Human Rights Related to Research Involving Human Subjects, Faculty of Medicine, Mahidol University; and the Ethical Review Committee for Research in Human Subjects, Ministry of Public Health of Thailand. Written informed consent was obtained from each patient prior to the study. The criteria of recruitment were patients aged over 40 years with a diagnosis of COPD based on post-bronchodilator ratio of FEV_1_/FVC < 0.7 in the past 6 months, smokers or ex-smokers with a smoking history of more than 10 pack-years, and no history of acute exacerbation for at least three months prior to the enrollment. Patients with a prior history of asthma were eligible if they were currently diagnosed with COPD. Subjects meeting any of the following criteria were not enrolled in the study: current diagnosis of asthma, current active respiratory disorders other than COPD, e.g. lung cancer, tuberculosis or other significant chest x-rays findings not associated with COPD (documented within 1 year). Those who were unable to complete questionnaires were also excluded.

### Study assessments and procedures

In order to assess the reliability and validity of the CAT questionnaires, the official Thai CAT questionnaire and St. George Respiratory Questionnaire (SGRQ) were administered at a single clinic visit. Demographic data and medical history were recorded. All subjects were assessed per routine practice: post-bronchodilator FEV_1_, FVC, % predicted of FEV_1_, and ratio of FEV_1_/FVC unless there were documented results of spirometry from the past 6 months. Values were calculated using NHANES (National Health and Nutrition Examination Survey) III reference equations [9]. However, for Asians, a correction factor of 0.88 was applied to the FVC and FEV_1_ predicted [10]. Body mass index (BMI) and MRC [11] were also assessed.

### Health status questionnaires

The Thai version of SGRQ and the official Thai CAT were administered to all subjects. The SGRQ is a 50-item questionnaire designed to measure the impact of COPD on the subject’s health-related quality of life (HRQoL). The questionnaire was self-completed by patients over an average of 20 minutes [12]. The original CAT designed to assess current health status has 8 items covering cough, phlegm, chest tightness, breathlessness, activity limitation, confidence, sleep, and energy. Each item is scored from 0 to 5 giving a total score ranging from 0 to 40, corresponding to the best and worst health status in patients with COPD, respectively [6]. The original CAT was translated to the official Thai through linguistic validation process undertaken by TransPerfect Translations, Inc. in 2009 in accordance with the International Society for Pharmacoeconomics and Outcome Research translation and cultural adaptation process for patient-reported outcomes [13]. The linguistic validation process involved both translation of the original English CAT to Thai (forward translation) and retranslation of the Thai CAT to English (backward translation). The process also included a reviewer to verify medical terms of the translation as well as a pilot test performed in 5 COPD patients. The final Thai CAT version was used for validation in this study.

### Statistical analysis

Data were expressed as mean ± SD unless otherwise stated. Comparisons of baseline characteristics between the COPD staging groups were performed using Fisher’s exact test for categorical variables. Continuous variables were analyzed using a one way ANOVA or Kruskal Wallis as appropriate. The internal consistency of the set of 8 items of Thai CAT questionnaire was estimated by applying Cronbach’s alpha coefficient and values > 0.70 were generally considered acceptable for aggregate data [14]. The correlation between CAT with SGRQ, FEV_1_, and MRC dyspnea scores were assessed using Pearson’s correlation. Statistical significance was accepted at p < 0.05 and summary results were presented as mean ± SD or N (%). All analyses were carried out with the SPSS statistical package, version 16 for Windows (SPSS Inc. IL, USA).

## Results

### Patients’ population

A total of 98 subjects from three hospitals were recruited in this study, comprising Maharaj Nakorn Chiang Mai Hospital (n = 40), Ramathibodi Hospital (n = 30), and Central Chest Institute of Thailand (n = 28). The mean age of the group was 71 years (52–89), 83% were male with 56.6 ± 20.9 % predicted of FEV_1_. Results of baseline characteristics between COPD severity stages were comparable. We found statistically significant differences among Global initiative for Chronic Obstructive Lung Disease (GOLD) in terms of age, smoking status, dyspnea score, lung function, and history of acute exacerbations. There were no significant differences among GOLD in terms of gender, BMI, pack-years smoking, and duration of disease and symptoms (Table 1).

**Table 1:** **Characteristics of the study population (n = 98)**

	COPD GOLD stage				***P-value***
	I (mild)	II (moderate)	III (severe)	IV (very severe)	
N	14	42	28	14	
Males	92.9	78.6	82.1	85.7	0.663
Age (years)	74.4 ± 8.8	70.8 ± 7.3	71.3 ± 8.5	64.9 ± 7.3	0.018
BMI (kg/m^2^)	22.9 ± 3.6	21.3 ± 3.7	20.6 ± 3.4	19.3 ± 3.1	0.061
Pack years	50.4 ± 46.5	38.2 ± 24.6	39.8 ± 41.5	57.0 ± 29.5	0.265
Current smoker	0.0	14.3	0.0	0.0	0.038
FEV_1_ (% predicted)	90.2 ± 7.4	65.2 ± 7.7	43.1 ± 5.3	24.4 ± 3.4	<0.001
FEV_1_/FVC (%)	62.5 ± 4.1	56.2 ± 9.4	44.9 ± 9.4	35.6 ± 7.5	<0.001
SGRQ total score	37.1 ± 20.7	36.9 ± 19.7	35.6 ± 20.9	50.7 ± 21.1	0.123
MRC dyspnea scale	1.8 ± 1.0	2.2 ± 0.9	2.5 ± 1.1	3.1 ± 1.2	0.015
Duration of COPD (months)					
By investigator report	62.5 ± 31.7	80.5 ± 75.9	76.5 ± 55.2	83.7 ± 51.3	0.788
Based on post-BD ratio FEV_1_/FVC < 0.7	56.8 ± 32.2	42.7 ± 36.3	49.2 ± 42.6	53.3 ± 37.3	0.600
Duration of chronic respiratory symptoms (months)					
Cough	48.4 ± 38.7	84.3 ± 97.4	76.1 ± 69.4	29.9 ± 96.7	0.584
Chronic dyspnea	54.8 ± 34.9	81.3 ± 96.4	74.7 ± 64.6	92.2 ± 74.6	0.644
History of AE in the last 12 month	14.3	14.3	32.1	71.4	<0.001
Number of AE in the last 12 month	0.2 ± 0.6	0.3 ± 1.2	0.5 ± 0.8	2.7 ± 4.4	<0.001
Family history of asthma	7.1	14.3	21.4	7.1	0.717

Data were mean ± SD or %, BMI: body mass index; FEV_1_ (% predicted): percentage predicted of forced expiratory volume in first second; FEV_1_/FVC: ratio of forced expiratory volume in first second to force vital capacity; SGRQ: St George’s respiratory questionnaire; MRC: Medical Research Council scale; AE: acute exacerbation.

### Validity

**Figure 1:**
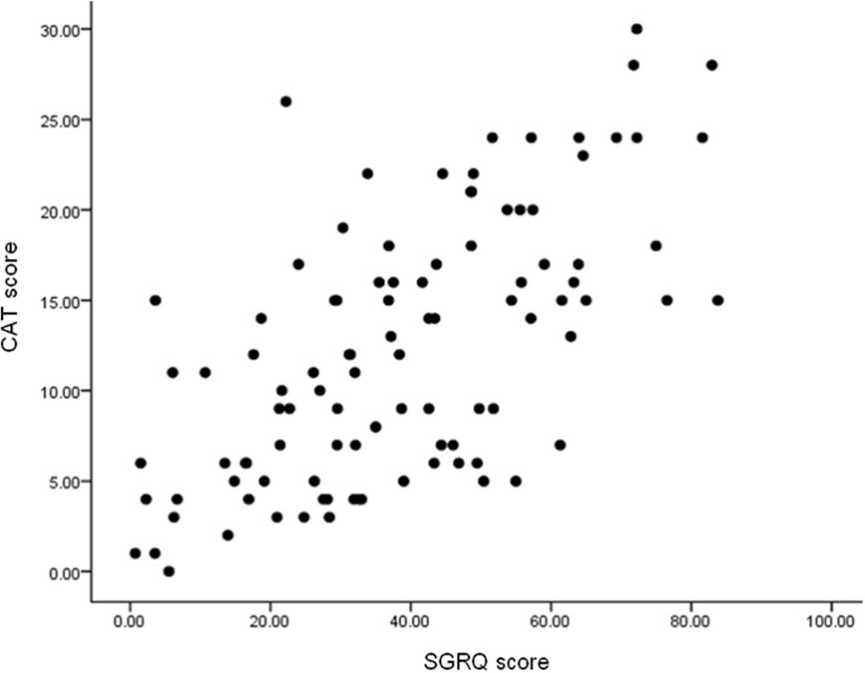
**Pearson correlation between scores in St George’s Respiratory Questionnaire (SGRQ) and COPD Assessment Test (CAT) in 98 patients.** r = 0.652, *p <0.001.*

Both Thai versions of SGRQ and CAT questionnaire were obtained from the same patients. The internal consistency of the CAT was high with Cronbach’s alpha = 0.853. Correlation between the CAT and the SGRQ was significant (r = 0.652, *p < 0.001*) for total scores (Figure 1). Correlations between the CAT and domains of SGRQ were also significant (r = 0.576, 0.566, and 0.591, *p < 0.001* for symptoms, activity, and impact domains, respectively) whereas the total score showed the best correlation. The correlation between MRC dyspnea score and CAT score was also significant (r = 0.550, *p < 0.001*) but not for the % predicted of FEV_1_ (r = − 0.193, *p = 0.109*) (Table 2).

**Table 2:** **Correlations between health status questionnaires (official Thai CAT and SGRQ), MRC and FEV**_**1**_

	CAT	SGRQ total	SGRQ symptom	SGRQ activity	SGRQ impact	MRC	%pred. of FEV _1_
CAT	1	0.652**	0.576**	0.566**	0.591**	0.550**	−0.193
SGRQ							
Total	0.652**	1	0.706**	0.882**	0.951**	0.546**	−0.135
Symptom	0.576**	0.706**	1	0.517**	0.600**	0.416**	−0.115
Activity	0.566**	0.882**	0.517**	1	0.725**	0.552**	−0.171
Impact	0.591**	0.951**	0.600**	0.725**	1	0.483**	−0.091
MRC	0.550**	0.546**	0.416**	0.552**	0.483**	1	−0.317**
%pred. Of FEV_1_	−0.193	−0.135	−0.115	−0.171	−0.091	−0.317**	1

**Correlations are significant at the 0.01 level. %pred. of FEV_1_: percent predicted of forced expiratory volume in first second; CAT: COPD assessment test; SGRQ: St George’s respiratory questionnaire; MRC: Medical Research Council scale.

### The CAT questionnaires-GOLD stage

**Figure 2:**
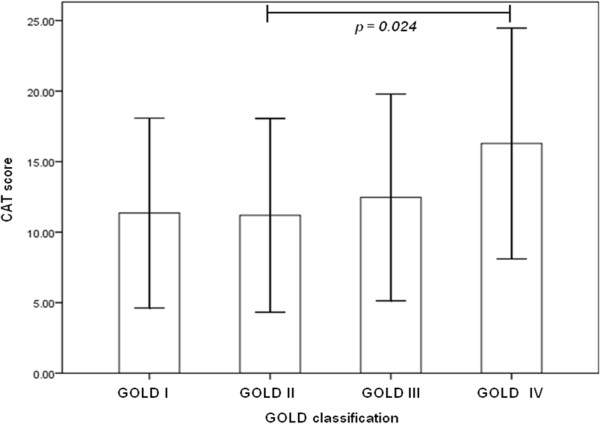
**COPD Assessment Test (CAT) scores for GOLD COPD stages.** Error bars represent means ± 1 SD. Numbers above line represent p values.

The comparison of mean CAT score and COPD severity stages (GOLD stages) revealed insignificantly different scores (11.3 ± 6.7, 11.2 ± 6.9, 12.5 ± 7.3 and 16.3 ± 8.2 for GOLD I, II, III, and IV, respectively, *p = 0.142*). However, patients with very severe COPD (GOLD IV) showed significantly higher CAT scores compared to the patients with moderate COPD (GOLD II) (*p = 0.024*) (Figure 2).

## Discussion

The CAT was designed to measure health status of COPD patients in clinical practice. It could be instantly calculated and was much shorter and easier to understand than the SGRQ, the standard assessment for COPD. Indeed, patients in a previous study reported that CAT was fairly easy, reflected well with their status, the response options, and rank systems [6]. Our study revealed that the official Thai CAT questionnaire had an internal consistency with a Cronbach’s alpha of 0.853 and was comparable to that of the original version of CAT (Cronbach’s a coefficient of 0.88) [6]. The validated Thai version of the SGRQ was used as an instrument to measure the validity of the official Thai CAT questionnaire. The result was significant, albeit moderate correlation supporting the validity of the official Thai CAT questionnaire (r = 0.652). This result was similar to other findings in previous studies which varied from moderate to high correlations with assessments using the COPD-specific St. George's Respiratory Questionnaire (SGRQ-C) (r = 0.64 to 0.82) [6, 15–17]. The CAT is not a diagnostic tool but could aid in the identification of the health impairment of COPD patients. Contrary to previous reports, our study found no significant difference of mean CAT scores across different GOLD stages (except GOLD II and IV) [15, 18] which might be due to the reason that FEV_1_ classified by GOLD stages in this study had poorest correlation with CAT (*r = −0.193*). The multiple consequences of COPD have no relationship with airflow limitation and as a result, FEV_1_ may not reflect the total impairment caused by the disease. This was expected as the pulmonary function measured by FEV_1_, on which the GOLD classification of COPD stage is based, is not a good predictor of health status [14]. MRC functional dyspnea is now proven to be useful in predicting outcomes in patients with COPD and is recommended in the routine handling and evaluation of these patients [19]. In the present study, we had the opportunity of testing the correlation between CAT and MRC dyspnea score. It was statistically significant with moderate correlation (r = 0.550) which is similar to other studies (0.50 to 0.579) [18, 20, 21]. Currently the most widely used questionnaire for measuring health status in COPD in a research setting is the SGRQ [22]. The main disadvantages for clinical practice are its extent as it comprises 50 questions which are viewed by the patients as rather complicated and time consuming and because scores can only be calculated using a computer-based system. On the other hand, CAT could be used as an easy and reliable tool to assess health status in COPD patients in clinical studies and might become a useful tool in routine clinical practice. Unlike the physician’s judgment of clinical severity, the CAT provides a standardized assessment and a numerical estimate of disease impact which is reported to be reliable across languages and countries [6, 16–18]. However, it is only one part of the clinician’s toolkit, to be used alongside spirometry, exacerbation history, and an assessment of co-morbidity. Its role is to supplement information obtained from lung function measurements and assessment of exacerbation risks. Like other clinical assessment techniques, its utility will only become fully apparent with time. This study provides the evidence that the official Thai CAT questionnaire is a reliable tool and is valid for providing practicing physicians a measurement of the impact of COPD on health status of their groups of patients. We need further studies to demonstrate its ability to reliably measure intra-individual change, detect interpretable differences to norms, and trigger or support useful discussion between patients and physicians which would contribute to better and more efficient care. Our study has a limitation in that it was a cross-sectional study based on a single visit; as a result repeatability estimated by calculating the intraclass correlation coefficient (ICC) was not analyzed. In addition, the validation findings were based on three sites of academic medical centers chosen on the basis of representing large referral centers for COPD patients: one site from northern part and two sites from central part of Thailand. Thus, the data might not be reliably extrapolated to all COPD populations in Thailand. Although the official Thai CAT questionnaire showed very similar properties to the much more complex Thai version of SGRQ, with a reliability of 0.853, it could not be recommended to be used in making important decisions about individuals [23]. However, it can be considered to use together with other clinical parameters to accurately measure the impact of COPD on patients’ health status.

## Conclusions

The official Thai CAT questionnaire is a reliable tool and has a good validity. It can be expected to serve as a short and simple questionnaire for assessment of the health status of Thai COPD patients.

## Abbreviations

COPD: Chronic obstructive pulmonary disease
CAT: COPD assessment test
QoL: Quality of life
HRQoL: Health-related QoL
SGRQ: St. George respiratory questionnaire
MRC: Medical Research Council Dyspnea Scale
GOLD: Global initiative for chronic obstructive lung disease
BMI: Body mass index
FEV1: Forced expiratory volume in first second
FVC: Forced vital capacity.
